# Systemic immune-inflammation index predicts survival in gastric cancer: a systematic review and meta-analysis

**DOI:** 10.3389/fonc.2026.1953559

**Published:** 2026-10-30

**Authors:** Ziyi Yu, Xue Liu, Guoliang Hu, Lingling Zhao, Tingting Liang

**Affiliations:** Department of Oncology, Jilin University First Hospital, Changchun, Jilin, China

**Keywords:** gastric cancer, meta-analysis, overall survival, prognostic biomarker, progression-free survival, recurrence-free survival, systemic immune-inflammation

## Abstract

**Objective:**

To comprehensively assess the association of pretreatment systemic immune-inflammation index (SII) with the prognosis of gastric cancer patients.

**Methods:**

PubMed, Web of Science, Embase, and the Cochrane Library were searched to select studies exploring the association of SII with the overall survival (OS), progression-free survival (PFS) or recurrence-free survival (RFS) of gastric cancer. We pooled the hazard ratios (HRs) with 95% confidence intervals (CIs) using a random-effects model. Robustness was assessed by sensitivity analyses, and publication bias was evaluated using funnel plots and Egger’s regression test.

**Results:**

A total of 61 studies involving 66 cohorts and 31,608 patients were included in this meta-analysis, with most studies adopting a retrospective design and only one prospective study enrolled. Elevated pretreatment SII was significantly correlated with inferior OS (HR = 1.48, 95% CI: 1.36–1.62), PFS (HR = 1.43, 95% CI: 1.30–1.58), and RFS (HR = 1.38, 95% CI: 1.09–1.76). Subgroup analyses demonstrated that the magnitude of the prognostic association varied across treatment modalities and geographic regions.

**Conclusion:**

In conclusion, we found that elevated pretreatment SII is a potential biomarker for adverse prognosis in gastric cancer patients. However, since nearly all included studies were retrospective and only one was a prospective study, our findings support the prognostic value of SII, but prospective validation is required before routine clinical implementation.

**Systematic review registration:**

https://www.crd.york.ac.uk/PROSPERO/, identifier CRD420261321880.

## 1 Introduction

Gastric cancer (GC) remains the fifth most common cancer in terms of global cancer burden and the fourth most common cause of cancer-related mortality. Based on the GLOBOCAN 2022 report published by the IARC in 2024, an estimated 969,000 individuals were newly diagnosed with gastric cancer and about 660,000 deaths occurred worldwide in 2022, which is 4.8% of total cancer diagnoses and 6.8% of cancer-related deaths (1). The burden of GC is mostly in East Asia, which accounts for 53.8% of global incident cases and 48.2% of global mortality (1). 60%-70% of Chinese GC patients are in locally advanced or metastatic stages at the time of initial presentation and miss the window of radical curative surgery (2). It is of critical clinical importance to identify inexpensive, reliable prognostic biomarkers for early risk stratification and to make the treatment decision for personalized treatment.

Chronic inflammation is believed to be involved in tumor initiation, progression and metastasis (3). cancer-related inflammation was first proposed as the seventh hallmark of cancer by Mantovani et al. in 2009 (4). In 2011, Hanahan and Weinberg classified the tumor promoting inflammation as one of the core enabling characteristics of malignancy (5). In the tumor microenvironment (TME), the peripheral blood inflammatory cells can tune the balance between pro-tumor inflammation and anti-tumor immunity: neutrophils and platelets are involved in the tumor invasion, angiogenesis and immune evasion (6, 7), while the lymphocytes are involved in mediating the cytotoxic anti-tumor responses (8). The balance directly affects tumor progression and the patient prognosis.

In this context, the composite inflammatory indices derived from the routine complete blood counts, namely the neutrophil-to-lymphocyte ratio (NLR), platelet-to-lymphocyte ratio (PLR) and the systemic immune-inflammation index (SII), have attracted considerable attention in cancer prognostication, because they are non-invasive, cheap and can be repeated easily (9). SII was first described by Hu et al. in 2014 as a prognostic biomarker of patients with hepatocellular carcinoma. It is calculated as: SII = (platelet count × neutrophil count)/lymphocyte count (10). Compared with the NLR and PLR, SII incorporates three important parts of systemic inflammatory and immune responses, which reflect the imbalance of the pro-tumor inflammatory process and the impaired anti-tumor immune function in a more comprehensive way. The prognostic value of SII has been confirmed in some kinds of solid tumors, such as the gastrointestinal malignancies (11).

In the field of GC, there are many single-center studies that have related the increased pretreatment SII to adverse clinicopathological features and poor survival (12–14). However, the findings in the studies are not consistent, because the sample size, the population of the patients, the treatment regimens and the cut-off of SII are all different. There were several meta-analyses that have tried to pool these data (15–17). The largest and most recent one was done by Yang and Wu in 2024, and it has 30 studies that confirmed higher pretreatment SII levels were significantly related to worse OS, (HR = 1.53, 95% CI: 1.34 -1.75) and RFS(HR = 1.41, 95% CI: 1.17 -1.70) in the people with GC (17).

However, the existing meta-analyses have critical unaddressed limitations (15, 17). First, most of the studies did not systematically assess the PFS, which is an important endpoint of the assessment of the treatment efficacy in advanced GC. Second, there is also a significant geographic bias, as the subgroup analyses of non-Chinese studies have wide confidence intervals and do not have enough statistical power. Third, the search cut-off dates of the previous review are relatively early, so the emerging data in 2024–2026 of SII in some novel treatment setting, including immune checkpoint inhibitors (ICIs) and perioperative chemotherapy, were not included. Accordingly, a revised and comprehensive systematic review and meta-analysis is needed. The present study was carried out to evaluate the association between the pretreatment SII and the survival outcomes (OS, PFS and RFS) in the GC. We systematically searched all the eligible studies published up to February 10, 2026 in PubMed, Embase, Web of Science and the Cochrane Library, and we performed the comprehensive subgroup analyses (stratified by treatment modality, geographic region, SII cut off value and treatment setting), sensitivity analyses, and publication bias assessment. This study was registered in PROSPERO (CRD420261321880) and we followed the PRISMA 2020 statement to evaluate the prognostic value of the SII in the GC.

## 2 Materials and methods

### 2.1 Literature search

We complied with the PRISMA 2020 statement throughout the study. The study protocol was registered in PROSPERO (CRD420261321880). The search strategy was developed by two investigators (YZY and LX). Subject headings and free-text keywords were independently designed for literature retrieval from PubMed, Web of Science, Embase, and the Cochrane Library. The search included studies published up to February 10, 2026 (**Figure 1**). We performed searches using terms related to systemic immune-inflammation index and gastric cancer. More details of the search strategy can be found in **Supplementary Table S1**.

**Figure 1:**
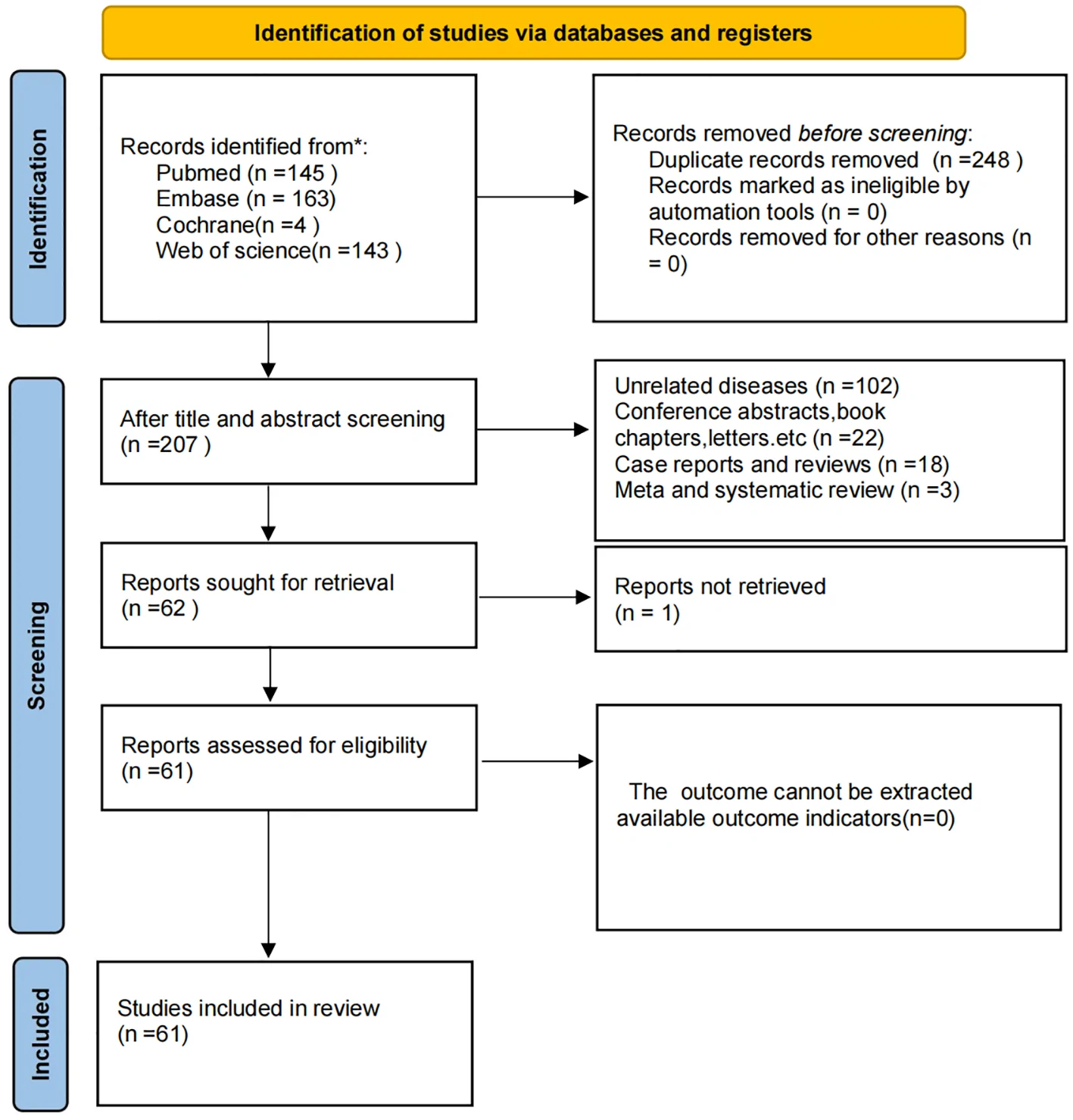
PRISMA 2020 flow diagram illustrating the study selection for this meta-analysis.

### 2.2 Study selection

Studies were included according to the criteria below: (1) individuals were diagnosed with GC based on pathological confirmation; (2) the studies analyzed the prognostic significance of SII for OS, PFS, or RFS; (3) hazard ratios (HRs) with 95% confidence intervals (CIs) were reported directly or could be calculated from the reported data; (4) participants were classified into high-and low-SII groups according to predefined cut-off values; and (5) the studies were fully published articles.

Studies were excluded if they met the following conditions: (1) reviews, comments, conference abstracts, case reports, letters, and other non-original articles; (2) studies not reporting enough data to obtain HRs and 95% CIs; (3) studies without survival outcome data; and (4) duplicate publications or studies with overlapping datasets.

Two investigators (YZY and LX) independently reviewed the titles and abstracts of the identified studies, reviewed the full texts, and assessed their eligibility for inclusion. In case of disagreement, they were discussed.

### 2.3 Data extraction

YZY and LX performed extraction independently. Any disagreements were resolved by consultation with all co-authors. The following data were extracted: the name of the first author, study location, publication year, study type, sample size, timing of SII assessment, cut-off value, treatment method, patient age and HRs for OS, RFS, and PFS. For studies in which HRs were calculated using the low-SII group as the reference, inverse HRs and corresponding CIs were computed, and the upper and lower CI limits were reversed to ensure consistency in the statistical analysis. Whenever multiple effect estimates were available for the same endpoint, multivariable-adjusted HRs were preferentially extracted, and unadjusted (univariate) HRs were used only when an adjusted estimate was not reported; because the covariates included in the adjusted models differed across studies, differences in adjustment status may contribute to between-study heterogeneity. Survival endpoints were recorded according to the terminology used in the original reports: PFS, DFS, and RFS are related but not fully interchangeable endpoints, and in surgical cohorts some endpoints originally labelled as PFS actually represent DFS or RFS. Endpoints were therefore assigned to the prespecified outcome categories (OS, PFS, and RFS) based on the definitions reported in each original article, and this classification may introduce a degree of clinical heterogeneity, which is acknowledged in the Discussion.

### 2.4 Quality assessment

We used the Newcastle-Ottawa Scale (NOS) for quality assessment across selection, comparability, and outcome domains; scores **≥ 7** indicated high quality.

### 2.5 Statistical analysis

HRs and their corresponding 95% CIs were pooled to evaluate the prognostic relevance of SII in gastric cancer. We assessed between-study heterogeneity using Cochran’s Q test and the I^2^ statistic. In view of the anticipated clinical and methodological diversity among the included studies, a random-effects approach was adopted for meta-analysis. The robustness of the pooled outcomes for OS, RFS, and PFS was further examined through sensitivity and subgroup analyses. Publication bias was investigated using funnel plots in combination with Egger’s regression test. P < 0.05 was considered statistically significant. All analyses were performed using Review Manager 5.4 and Stata 18.0.

## 3 Results

### 3.1 Study characteristics

In total, 455 records were identified by database searches. After removing 248 duplicate entries, 207 studies were left for subsequent title and abstract review. Of these records, 145 were excluded due to unrelated diseases (n = 102), conference abstracts, book chapters, or letters (n = 22), case reports or review articles (n = 18), and previous meta-analyses or systematic reviews (n = 3). Subsequently, 62 full-text reports were identified for retrieval, but 1 report could not be obtained. Eligibility screening was performed for the remaining 61 reports, and none were excluded owing to unavailable outcome indicators. Finally, 61 eligible studies were included in the quantitative synthesis. In contrast, Liu 2015 and Huang 2016 (both n = 455) were verified as independent cohorts according to their distinct enrollment periods and SII cut-offs, as were Shi 2018 and Shi 2022; both pairs were therefore retained (9, 13, 14, 18–76). Furthermore, we extracted two independent cohorts from some studies (22, 31, 38, 39, 43). Data from 31,608 patients were analyzed. The available evidence was largely from studies conducted in China. All included studies were published in English. Except for one prospective study, the remaining studies were retrospective. Therefore, data for OS, PFS, and RFS were available from 60, 20, and 7 cohorts, respectively (**Table 1**).

**Table 1:** Basic characteristics of the included literature.

Author	Year	Country	Study design	Age (median/mean)	Enrollment period	Sample size	SII cut-off	Surgery	Neoadjuvant therapy	Outcome	NOS score	Immunotherapy
Liu	2015	China	Retrospective	59	2005-2010	455	660	Yes	No	OS	8	No
Huang	2016	China	Retrospective	56	2013-2014	455	572	Yes	No	OS	8	No
Chen	2017	China	Retrospective	57	2007-2015	292	600	Yes	Yes	OS,PFS	7	No
Wang	2017	China	Retrospective	56	1994-2005	444	660	Yes	No	OS	8	No
Shi	2018	China	Retrospective	56	2012-2014	862	320	Yes	No	OS	7	No
Lu	2019	China	Retrospective	NA	2015-2016	401	784.7	Yes	No	RFS	7	No
Wang	2019	China	Retrospective	55.7	2009-2012	182	600	Yes	No	OS,PFS	8	No
Gu	2020	China	Retrospective	61.7	2013-2016	598	566	Yes	No	PFS	8	No
Yin	2020	China	Retrospective	56	2004-2014	278	524.45	Yes	No	OS	8	No
Zhu	2020	China	Retrospective	55	2001-2013	512	527	Yes	No	OS	7	No
Chen	2021	China	Retrospective	60	2015-2019	139	665.3	No	No	OS,PFS	8	Yes
Hirahara	2021	Japan	Retrospective	70	2010-2017	415	661.9	Yes	No	OS	8	No
Inoue	2021	Japan	Retrospective	67	2008-2013	447	395	Yes	No	OS,RFS	7	No
Jomrich	2021	Austria	Retrospective	67.2/63.7	1992-2016	320	644	Yes	Yes/No	OS,PFS	8	No
Lin	2021	China	Retrospective	60.9	2009-2014	2,257	569.93	Yes	No	OS	7	No
Wang	2021	China	Retrospective	61	2012-2016	608	372.8	Yes	No	OS	7	No
Cavdar	2022	Turkey	Retrospective	67	2012-2022	216	880.34	No	No	OS	8	No
He	2022	China	Retrospective	61	2009-2016	358	508.3	Yes	No	OS	7	No
Li	2022	China	Retrospective	54.46	2012-2019	223	290.5	Yes	No	OS	8	No
Liang	2022	China	Retrospective	61	2010-2019	2,443	962.5	Yes	No	OS	8	No
Qu	2022	China	Retrospective	NA	2019-2021	106	1140.91	No	No	OS,PFS	7	Yes
Shi	2022	China	Retrospective	NA	2011-2017	675	315	Yes	No	OS	8	No
Wang	2022	China	Retrospective	NA	2010-2018	89	369.2	Yes	No	PFS	8	No
Wei	2022	China	Retrospective	59	2019-2021	218	1185.2	Yes	No	OS	8	No
Xu	2022	China	Retrospective	59.3	2010-2018	354	489.52	Yes	No	OS,PFS	8	No
Yekedüz	2022	Turkey	Retrospective	53/58	2004-2019	291	708	Yes	No	RFS	7	No
Yuan	2022	China	Retrospective	59	2016-2021	357	NA	No	No	OS	7	Yes
Chen	2023	China	Retrospective	NA	2014-2019	812	540	No	No	OS	8	No
Demircan	2023	Turkey	Retrospective	60	2014-2021	150	741	Yes	Yes	OS,PFS	8	No
Hannarici	2023	Turkey	Retrospective	64	2014-2021	159	774.7	No	No	OS,PFS	8	No
He	2023	China	Retrospective	63	2019-2022	158	823	No	No	OS,PFS	7	Yes
Liu a	2023	China	Retrospective	63.99	2014-2016	238	915.48	Yes	No	OS	7	No
Liu b	2023	China	Retrospective	59.43	2012-2018	1,133	712.58	Yes	No	OS	8	No
Luo	2023	China	Retrospective	NA	2010-2013	543	457.6	Yes	No	OS	7	No
Nechita	2023	Romania	Retrospective	64.85	2016-2021	549	753.88	Yes	Yes	OS	7	No
Shen	2023	China	Retrospective	54	2016-2018	330	388.5	Yes	No	OS	8	No
Ucar	2023	Turkey	Retrospective	65.9	2015-2022	103	637	Yes	No	OS	7	No
Uzunoglu	2023	Turkey	Retrospective	63.8	2012-2018	152	892	Yes	No	OS	7	No
Wang	2023	China	Retrospective	58	2013-2014	542	489.9	Yes	No	OS,RFS	8	No
Yang	2023	China	Prospective	62.7	2017-2022	102	448.1/376.9	No	No	OS,PFS	7	No
Zheng	2023	China	Retrospective	61	2010-2017	2,875	547.1	Yes	No	OS	8	No
Aoyama	2024	Japan	Retrospective	70	2005-2020	258	550	Yes	No	OS,RFS	8	No
Ba	2024	China	Retrospective	59.05	2016-2019	291	533.41	Yes	No	OS,PFS	8	No
Chen	2024	China	Retrospective	56	2014-2021	128	465.42	Yes	No	OS	7	No
Dong	2024	China	Retrospective	62	2020-2022	197	905.17	No	No	OS	7	Yes
He	2024	China	Retrospective	63.18	2016-2020	242	718.025	Yes	No	OS	8	No
Jing	2024	China	Retrospective	58.9	2013-2018	490	613.05	Yes	No	PFS	8	No
Wang	2024	China	Retrospective	NA	2012-2017	256	629.5	Yes	No	OS	7	No
Zeng	2024	China	Retrospective	NA	2010-2017	730	456.3	Yes	No	OS,PFS	8	No
Zhang	2024	China	Retrospective	NA	2013-2022	75	755.75	Yes	No	OS	7	No
Zhu	2024	China	Retrospective	NA	2019-2022	88	814.8	No	No	OS,PFS	7	Yes
Akdogan	2025	Turkey	Retrospective	60	2014-2020	74	681	Yes	Yes	OS,RFS	7	No
Du	2025	China	Retrospective	58	2016-2018	1,101	1281.88	Yes	No	OS	7	No
Guliyev	2025	Turkey	Retrospective	59	2011-2023	203	105.01	No	No	OS	8	No
Liu	2025	China	Retrospective	58.14	2012-2016	4,049	427.46	Yes	No	OS	7	No
Mildanoglu	2025	Turkey	Retrospective	58	2021-2024	153	1001.2	No	No	OS,PFS	8	Yes
Peker	2025	Turkey	Retrospective	64	2019-2025	168	685	Yes	Yes	OS	8	No
Shen	2025	China	Retrospective	64	2018-2021	299	1127	Yes	Yes	OS,PFS	8	Yes
Sun	2025	China	Retrospective	NA	2018-2023	142	548.22	No	No	OS	8	Yes
Udagawa	2025	Japan	Retrospective	66	2017-2023	277	1810	No	No	OS	7	Yes
Xie	2025	China	Retrospective	55.6	2011-2018	546	720.8	Yes	No	OS	7	No

NOS, Newcastle-Ottawa Scale; SII, systemic immune-inflammation index; OS, overall survival; PFS, progression-free survival; RFS, recurrence-free survival. NA, not available, indicates that relevant data were not reported in the original articles.

### 3.2 Study quality

The included studies achieved NOS scores of 7 -8, which reflected relatively high methodological quality across all 61 studies (**Supplementary Table S2**).

### 3.3 Meta-analysis results

#### 3.3.1 SII and OS

We evaluated the association between pretreatment SII and OS across 60 cohort studies. Given the considerable heterogeneity observed (Tau^2^ = 0.08; P < 0.00001; I^2^ = 87%), a random-effects model was employed. The pooled results indicated a statistically significant relationship between high pretreatment SII levels and reduced OS in gastric cancer patients (HR = 1.48; 95% CI: 1.36 -1.62; P < 0.00001), so individuals with high pretreatment SII levels had a 48% higher risk of all-cause mortality compared with those with low SII levels (**Figure 2a**).

**Figure 2:**
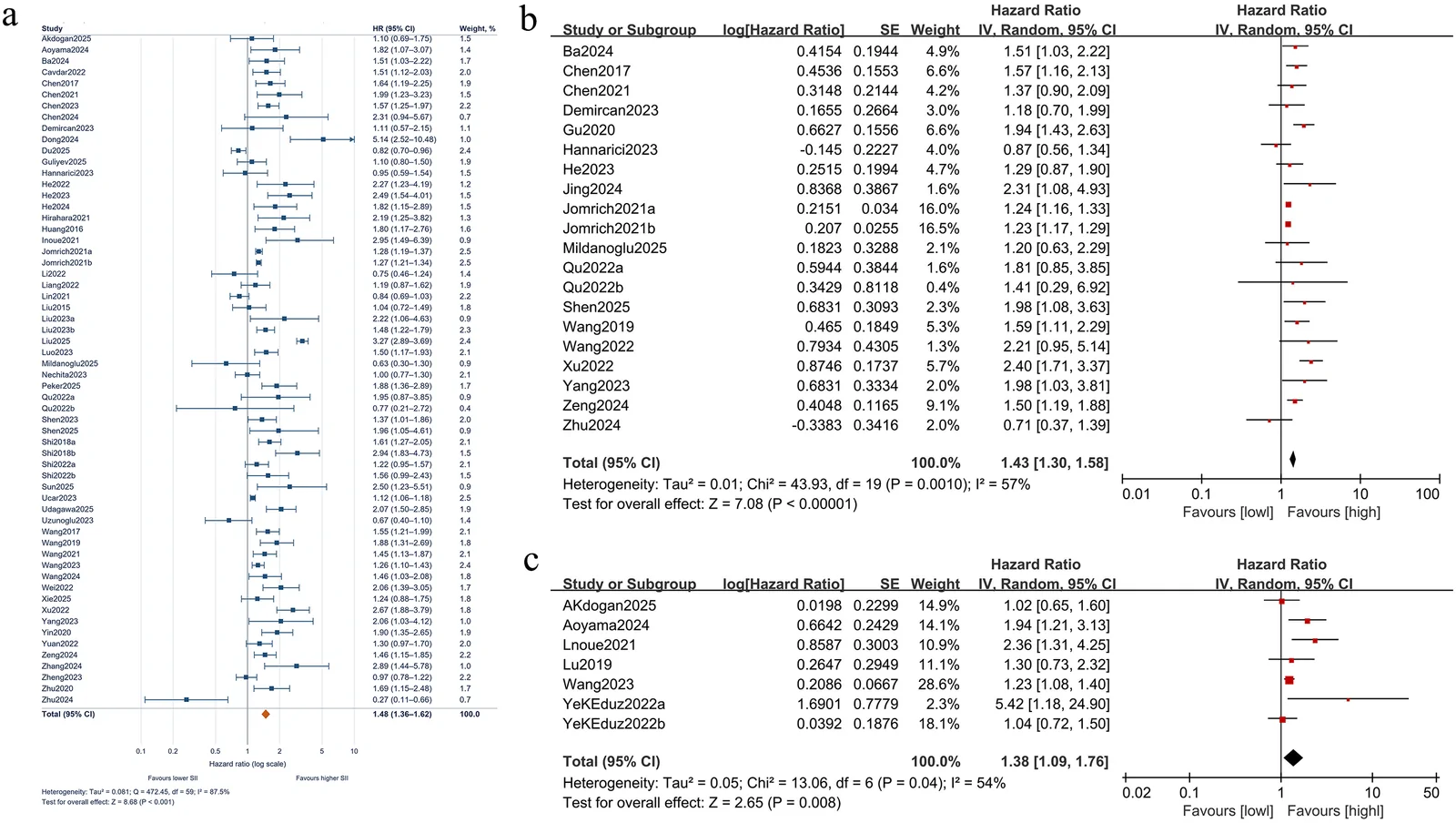
**(a)** Forest plot of the association between pretreatment SII and overall survival in gastric cancer. **(b)** Forest plot of the association between pretreatment SII and progression-free survival in gastric cancer. **(c)** Forest plot of the association between pretreatment SII and recurrence-free survival in gastric cancer.

We further examined potential contributors to heterogeneity through subgroup analyses (**Supplementary Table S3**). When stratified by treatment modality, we found comparable prognostic effects in the surgical group (n = 45; HR = 1.48; 95% CI: 1.34 -1.63; I^2^ = 89%) and the non-surgical group (n = 15; HR = 1.51; 95% CI: 1.20 -1.90; I2 = 74%). Furthermore, among surgical cohorts with available neoadjuvant data, heterogeneity markedly decreased in the neoadjuvant therapy subgroup (n = 7; HR = 1.33; 95% CI: 1.12 -1.57; I2 = 48%) compared with the direct surgery subgroup (n = 38; HR = 1.50; 95% CI: 1.34 -1.69; I2 = 91%). Stratification by immunotherapy status demonstrated that the prognostic association continued to be significant in both the immunotherapy subgroup (n = 11; HR = 1.61; 95% CI: 1.13 -2.29; P = 0.008; I^2^ = 77%) and the non-immunotherapy subgroup (n = 49; HR = 1.46; 95% CI: 1.33 -1.60; P < 0.00001; I2 = 89%). Age-based analysis indicated significant associations in both older cohorts (>60 years: n = 23; HR = 1.38; 95% CI: 1.25 -1.53; I2 = 80%) and younger cohorts (≤60 years: n = 26; HR = 1.51; 95% CI: 1.25 -1.81; I2 = 91%). Similarly, analysis by SII cut-off value demonstrated significant prognostic effects in both the higher cut-off subgroup (>600: n = 31; HR = 1.36; 95% CI: 1.24 -1.49; I2 = 79%) and the lower cut-off subgroup (≤600: n = 28; HR = 1.62; 95% CI: 1.37 -1.92; I2 = 89%). Sample size stratification also yielded significant associations in both large studies (>450: n = 18; HR = 1.34; 95% CI: 1.08 -1.65; P = 0.007; I^2^ = 94%) and smaller studies (≤450: n = 42; HR = 1.56; 95% CI: 1.43 -1.71; I2 = 77%) (**Supplementary Table S3**).

Geographic subgroup analysis demonstrated that the prognostic association was significant across most regions, including Chinese cohorts (n = 44; HR = 1.58; 95% CI: 1.38 -1.81; I^2^ = 88%), Japanese (n = 4; HR = 2.11; 95% CI: 1.67 -2.67; I2 = 0%), and Austrian cohorts (n = 2; HR = 1.27; 95% CI: 1.22 -1.33; I^2^ = 0%). The Turkish subgroup indicated a marginal association (n = 9; HR = 1.14; 95% CI: 0.96 -1.35; P = 0.14; I2 = 55%). By contrast, the single Romanian cohort did not show a significant association (HR = 1.00; 95% CI: 0.77 -1.30; P = 0.99). These findings suggest that treatment approach, immunotherapy administration, age, SII cut-off, sample size, and regional differences in patient characteristics may modulate the magnitude of the prognostic association, rather than being definitively established sources of heterogeneity. The forest plot of the individual study estimates and the pooled effect is shown in **Figure 2a**.

#### 3.3.2 SII and PFS

We found 20 cohort studies with data on the association between pretreatment SII and PFS. Because of the moderate heterogeneity across the pooled studies (I^2^ = 57%), the random effects model was used. The pooled analysis found that the elevated baseline SII was a predictor of poorer PFS (HR = 1.43; 95% CI: 1.30 -1.58; P < 0.00001). This means that, among the individuals with high pretreatment SII levels, the risk of progression or death was 43% higher than the participants with lower pretreatment SII levels.

To find sources of this variability, we performed subgroup analyses (**Supplementary Table S3**). When stratified by treatment approach, we found decreased heterogeneity in the non-surgical group (n = 8; HR = 1.22; 95% CI: 0.98 -1.52; I^2^ = 17%); but no association was statistically significant in this subgroup (P = 0.08). On the contrary, in the surgical group, the association was significant (n = 12; HR = 1.50; 95% CI: 1.34 -1.68; P < 0.00001; I^2^ = 69%). Among surgical cohorts, heterogeneity was further decreased in the neoadjuvant therapy subgroup (n = 4; HR = 1.35; 95% CI: 1.13 -1.60; I^2^ = 33%) in comparison with the direct surgery (n = 8; HR = 1.68; 95% CI: 1.35 -2.08; I^2^ = 77%). When we analyzed by geography, we found the effect is consistent in Chinese cohorts (n = 15; HR = 1.64; 95% CI: 1.45 -1.85; I^2^ = 18%), but not in Turkish studies (n = 3; HR = 1.03; P = 0.87) (**Supplementary Table S3**). Subgroup analyses by immunotherapy status, age, and sample size, gave consistent significant associations in all strata, and we found very low heterogeneity in the immunotherapy subgroup (I^2^ = 0%). These results indicate that elevated baseline SII is consistently associated with worse PFS in most of the clinical contexts, but SII cut-off selection, treatment approach and regional differences may modulate the prognostic value of SII. **Figure 2b** shows the individual study estimates and the pooled result.

#### 3.3.3 SII and RFS

A total of 7 cohort studies reported on the prognostic impact of pretreatment SII on RFS. Given moderate heterogeneity across the pooled studies (Tau^2^ = 0.05; chi^2^ = 13.06, df = 6; P = 0.04; I^2^ = 54%), a random-effects model was employed. The pooled analysis supported a significant relationship between increased baseline SII and worse RFS (HR = 1.38; 95% CI: 1.09 -1.76; P = 0.008), indicating that individuals with high pretreatment SII levels had a 38% increased risk of recurrence or death compared with those with low SII levels. The number of available studies was too small for subgroup analyses. Visual inspection of the forest plot showed that the observed heterogeneity might be partially attributed to variations in effect magnitude across individual studies, with one study reporting a relatively extreme effect estimate (Yekedüz 2022: HR = 5.42; 95% CI: 1.18 -24.90). Examination of the funnel plot showed reasonable symmetry, suggesting no substantial publication bias. The individual study estimates and pooled result are illustrated in **Figure 2c**.

### 3.4 Sensitivity analysis

Sensitivity analyses were performed to assess the robustness of the pooled results through stepwise omission of each study. For OS, when we omitted sequentially each of the 60 cohorts we obtained pooled HRs ranging from 1.43 to 1.50, with all 95% CIs excluding the null value (**Figure 3a**). For PFS, when we omitted each of the 20 cohorts, the pooled HRs ranged from 1.36 to 1.50, and all resultant 95% CIs remained above 1.0 (**Figure 3b**). For RFS, sequential omission of each of the 7 cohorts yielded pooled HRs ranging from 1.28 to 1.50, with all 95% CIs excluding the null value (**Figure 3c**). These results suggest that the pooled estimates for OS, PFS, and RFS were not strongly affected by any individual study.

**Figure 3:**
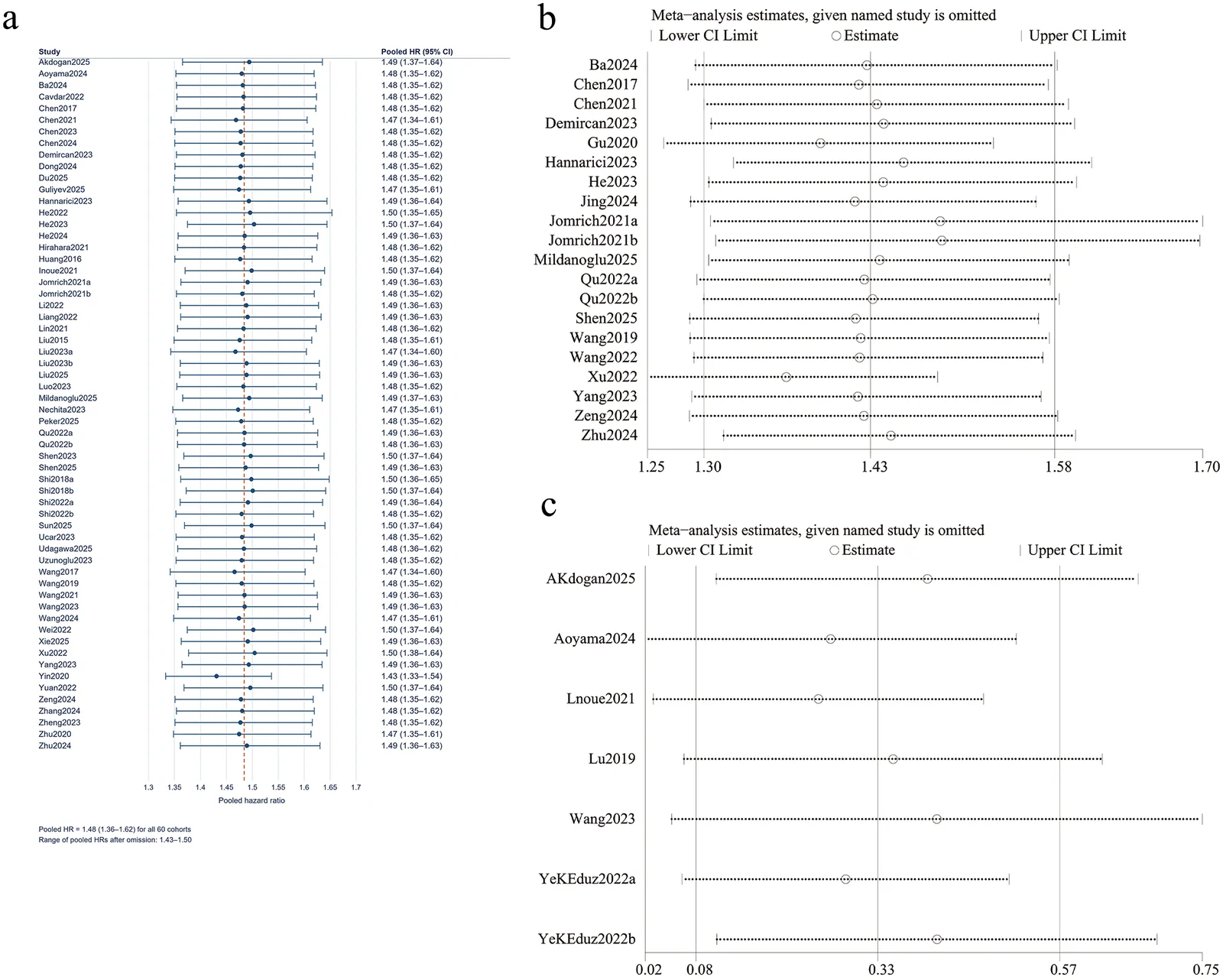
**(a)** Sensitivity analysis of the association between pretreatment SII and overall survival in gastric cancer. **(b)** Sensitivity analysis of the association between pretreatment SII and progression-free survival in gastric cancer. **(c)** Sensitivity analysis of the association between pretreatment SII and recurrence-free survival in gastric cancer.

### 3.5 Publication bias

For the three survival outcomes, potential publication bias and small-study effects were investigated by visual examination of funnel plots and Egger’s linear regression test. For OS, visual inspection of the funnel plot showed asymmetry, with relatively few small studies showing null or protective effects. The presence of significant small-study effects was also confirmed by Egger’s regression test (P = 0.030, **Figures 4a**, **5a**). For PFS, the funnel plot displayed noticeable asymmetry consistent with potential publication bias, and Egger’s test indicated statistical significance (P = 0.018, **Figure 4b**, **5b**). These findings suggest that the published literature may disproportionately consist of studies with positive or significant findings, which could lead to an overestimation of the pooled effect sizes for OS and PFS. For RFS, however, the funnel plot appeared reasonably symmetric upon visual inspection, and Egger’s test provided no evidence of significant effects (P = 0.197, **Figures 4c**, **5c**), indicating that publication bias is unlikely to have substantially influenced the RFS estimate. Nevertheless, because of the small number of included cohorts (n = 7), the statistical power of this assessment is limited, and the null finding should be interpreted with caution. Given that publication bias was detected for OS and PFS, the pooled HRs for these endpoints should be interpreted cautiously. Of note, funnel-plot asymmetry may also arise from true heterogeneity rather than publication bias alone.

**Figure 4:**
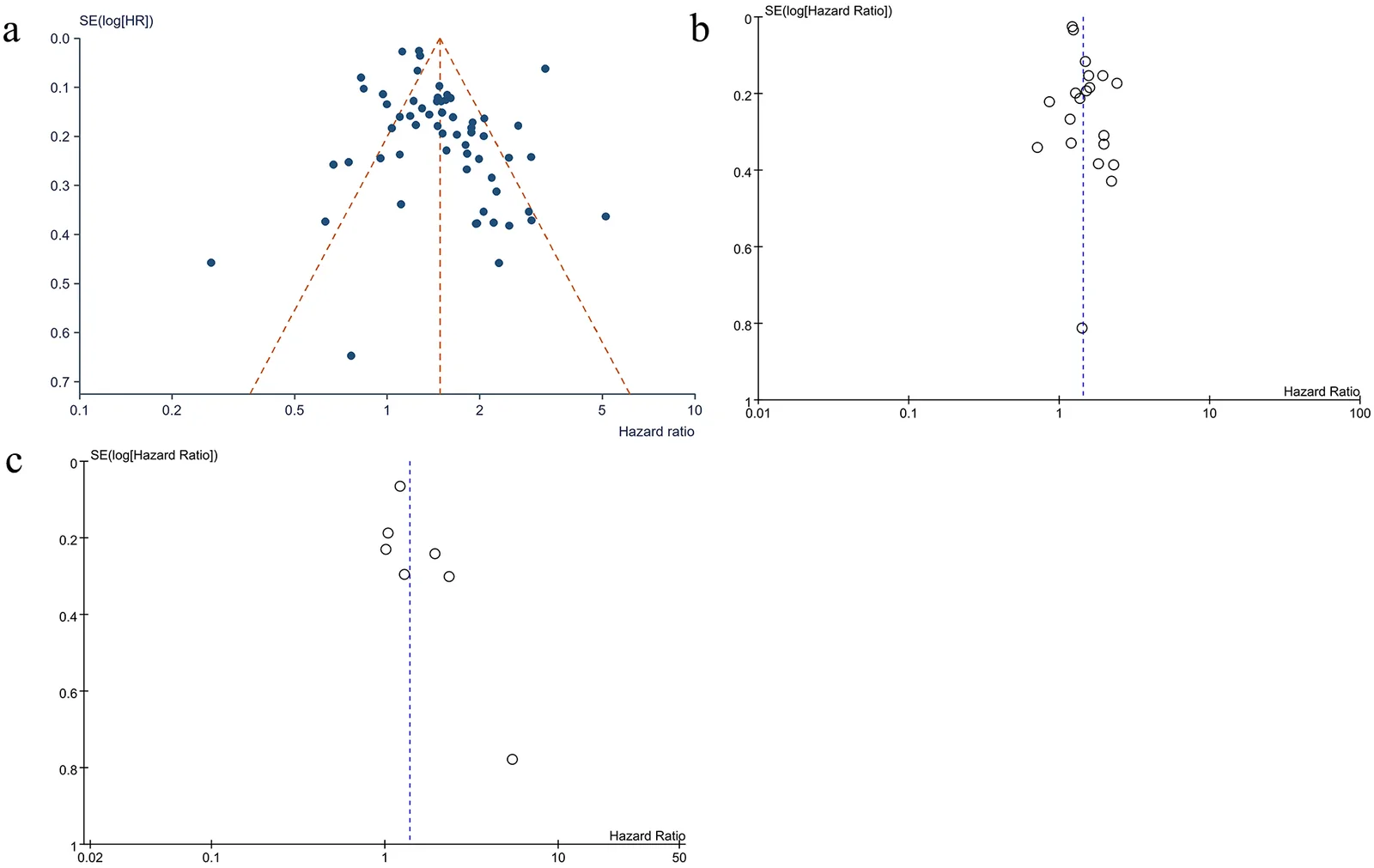
**(a)** Funnel plot for assessing publication bias in the analysis of pretreatment SII and overall survival. **(b)** Funnel plot for assessing publication bias in the analysis of pretreatment SII and progression-free survival. **(c)** Funnel plot for assessing publication bias in the analysis of pretreatment SII and recurrence-free survival.

**Figure 5:**
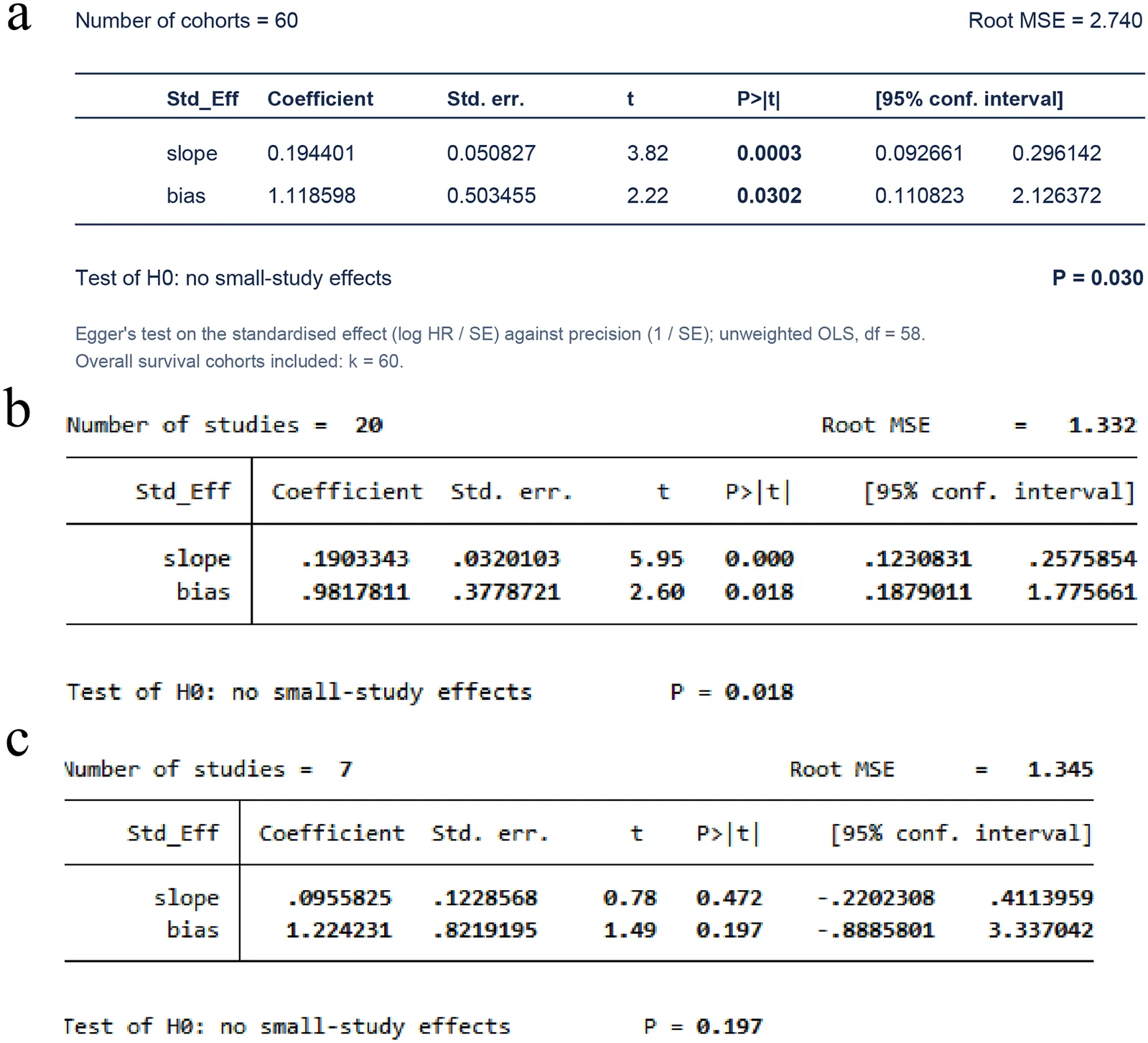
**(a)** Egger’s regression test for publication bias in the analysis of pretreatment SII and overall survival. **(b)** Egger’s regression test for publication bias in the analysis of pretreatment SII and progression-free survival. **(c)** Egger’s regression test for publication bias in the analysis of pretreatment SII and recurrence-free survival.

## 4 Discussion

Inflammation is a core driver of tumorigenesis and tumor progression, influencing gene mutations, genomic instability, as well as tumor proliferation and metastasis. Composite inflammatory indices based on routine complete blood counts provide a convenient and reproducible approach to assess systemic inflammatory status, and have suggested significant prognostic value across multiple solid tumors. The systemic immune-inflammation index (SII) integrates information from three peripheral blood cell types -neutrophils, platelets, and lymphocytes. Compared with single cell-based ratios, SII can more comprehensively reflect the imbalance between pro-tumor inflammation and anti-tumor immunity.

Overall, 61 studies comprising 66 cohorts and 31,608 patients were ultimately analyzed. The results showed a significant link between elevated pretreatment SII and OS (HR = 1.48, 95% CI: 1.36 -1.62), PFS (HR = 1.43, 95% CI: 1.30 -1.58), and RFS (HR = 1.38, 95% CI: 1.09 -1.76). Subgroup analyses suggested that the prognostic performance of SII varied across treatment modalities and geographic regions: heterogeneity of OS in the neoadjuvant therapy subgroup was markedly lower than that in the direct surgery subgroup (I^2^ = 55% vs.91%), the Japanese cohorts had the strongest effect size for OS (HR = 2.11), whereas PFS showed no significant association in the Turkish cohort. These discrepancies may reflect differences in tumor biology, treatment regimens, and host immune backgrounds across regions.

The meta-analysis done by Yang and Wu (2024) included 30 studies and reported that elevated SII was linked to a poor OS and RFS in gastric cancer. Compared with the above-mentioned previous work, the present study more than doubled the number of included studies, systematically evaluated PFS in addition to OS and RFS, and extended the literature search to up to February 10, 2026 to incorporate data from novel treatment modalities including immune checkpoint inhibitors (ICIs) and perioperative chemotherapy, thus providing updated evidence that may inform future prospective evaluation of SII in contemporary gastric cancer management.

The prognostic value of SII is supported by its underlying biological rationale: neutrophils and platelets promote tumor progression through multiple mechanisms, including the release of pro-angiogenic factors, formation of neutrophil extracellular traps (NETs), and facilitation of circulating tumor cell survival, whereas lymphopenia reflects impaired anti-tumor immune function. Notably, current evidence suggests that SII may serve not only as a baseline prognostic biomarker but also as a dynamic monitoring tool, given that dynamic changes in SII during treatment have been associated with treatment response and survival in gastric cancer. Supporting this concept, dynamic changes in SII during neoadjuvant chemotherapy have been shown to predict pathological tumor response and overall survival in patients with gastric or gastroesophageal junction cancer (45). These findings support the broader rationale that serial SII assessment may capture clinically meaningful information beyond a single pretreatment measurement, although prospective evidence in the immunotherapy setting is still limited. However, the exact mechanistic role of SII in gastric cancer needs to be further clarified.

The present study has several limitations. First, most included studies were retrospective, and only one prospective study was included, so the possibility of selection bias and unidentified confounding cannot be excluded. Second, the SII cut-off values varied substantially across the included studies (ranging from 105 to 1,810; **Table 1**), which precludes the establishment of a standardized prognostic threshold and may limit the direct clinical applicability of SII. Notably, our subgroup analysis suggested a stronger prognostic effect in cohorts using lower cut-offs (≤600: HR = 1.63) than in those using higher cut-offs (>600: HR = 1.36), indicating that threshold selection may modulate the magnitude of the prognostic association rather than merely adding statistical noise. Until an optimal, setting-specific cut-off is defined through prospective studies, the pooled estimates derived from dichotomized SII should be interpreted with caution and should not be extrapolated to continuous SII values. Third, evidence of publication bias was identified in the OS and PFS analyses. Fourth, the RFS analysis included only 7 studies, rendering this endpoint less robust. Fifth, methodological quality was assessed with the Newcastle-Ottawa Scale, which was designed for cohort and case-control studies rather than specifically for prognostic factor studies; a dedicated tool such as QUIPS may have provided a more appropriate risk-of-bias assessment. Sixth, the certainty of the evidence was not formally evaluated using the GRADE approach. In addition, the included studies were predominantly performed in Asian populations, and caution is warranted when extrapolating the conclusions to other ethnic groups and geographic regions.

These findings should be further confirmed by future prospective studies with standardized SII cut-off values. Further, investigations about the dynamic monitoring of SII during treatment, particularly in the context of immunotherapy and perioperative chemotherapy, may further refine its clinical utility. Establishing optimal SII thresholds across different treatment modalities and ethnic populations represents a critical next step toward personalized prognostic stratification in gastric cancer.

## 5 Future research directions

Prospective validation studies: Most included studies were retrospective, and only one prospective study was included. Validation of the independent prognostic value of SII will require future large -scale, multicenter prospective cohort studies, together with efforts to reduce selection bias and confounding factors and to strengthen the reliability and generalizability of the evidence.

Establishment of standardized cut-off values: The SII cut-off values adopted across studies are very different, representing an important source of heterogeneity. Future research should determine optimal cut-offs for different treatment settings and populations using large-sample data, and explore standardized protocols for clinical application.

Dynamic monitoring and treatment guidance: Existing studies have largely focused on baseline SII. Future investigations should examine the prognostic implications of dynamic SII changes during treatment-particularly in the context of immune checkpoint inhibitor and the chemotherapy in the perioperation -and assess its feasibility as a treatment response monitoring tool.

Cross-ethnic and cross-regional validation: The current evidence comes predominantly from Asian populations, with regional differences in the strength of the association: Japanese cohorts showed the strongest and most homogeneous effect for OS (HR = 2.11; I2 = 0%), whereas Turkish cohorts showed weaker and non-significant associations. Variations in SII cut-offs among studies could also account for inconsistent regional hazard ratio estimates. Future studies should validate the prognostic significance of SII in European, American, African, and other populations to clarify the impact of ethnic and geographic variation.

Mechanistic elucidation and combined model construction: It is the task of future studies to clarify the molecular mechanisms by which SII influences gastric cancer progression, and explore the integration of SII with tumor stage, molecular biomarkers, or other inflammatory indices to construct a more precise personalized prognostic assessment system.

## 6 Conclusion

This meta-analysis (66 cohorts from 61 studies; n = 31,608) shows that patients with high pretreatment SII do have worse OS, PFS, and RFS in gastric cancer. However, the magnitude of the prognostic association varied across treatment modalities and geographic regions. With the current evidence, which is not enough for the direct use in clinic due to the mostly retrospective designs, the heterogeneous cut-offs and the publication bias, more standardized prospective studies should be done to confirm the SII as a routine prognostic biomarker and think about its use in the dynamic monitoring.

## Data Availability

The original contributions presented in the study are included in the article/**Supplementary Material**. lFurther inquiries can be directed to the corresponding authors.
