# Supplementary material for: Systemic immune-inflammation index predicts survival in gastric cancer: a systematic review and meta-analysis
Source: Front Oncol. 2026 Oct 30;16:1953559. doi: 10.3389/fonc.2026.1953559 (PMC13759391; doi:10.3389/fonc.2026.1953559)
Supplement: Supplementary file 1 [file Table2.docx]

| Supplementary Table S3. Quality evaluation of the eligible studies with Newcastle–Ottawa scale. | | | | | | | | | |
| --- | --- | --- | --- | --- | --- | --- | --- | --- | --- |
| Study | Selection | | | | Comparability | | Outcome | | |
|  | Representative-ness | Selection of  non-exposed | Ascertainment  of exposure | Outcome not present at start | Comparability on most important factors | Comparability on other risk factors | Assessment of outcome | Long enough follow-up (median≥1 year) | Adequacy  (completeness) of follow-up |
| Liu2015 | * | * | * | * | - | * | * | * | * |
| Huang20126 | * | * | * | * | * | - | * | * | * |
| Chen2017 | * | * | * | * | - | - | * | * | * |
| Wang2017 | * | * | * | * | * | - | * | * | * |
| Shi2018 | * | * | * | * | - | * | - | * | * |
| Lu2019 | * | * | * | * | * | - | - | * | * |
| Wang2019 | * | * | * | * | - | * | * | * | * |
| Gu2020 | * | * | * | * | * | - | * | * | * |
| Li2020 | * | * | * | * | * | - | * | * | * |
| Yin2020 | * | * | * | * | - | * | * | * | * |
| Zhu2020 | * | * | * | * | * | - | - | * | * |
| Chen2021 | * | * | * | * | - | * | * | * | * |
| Hirahara2021 | * | * | * | * | * | - | * | * | * |
| Inoue2021 | * | * | * | * | - | - | * | * | * |
| Jomrich2021 | * | * | * | * | - | * | * | * | * |
| Lin2021 | * | - | * | * | * | - | * | * | * |
| Wang2021 | * | * | * | * | * | - | * | - | * |
| Yin2021 | * | * | * | * | - | - | * | * | * |
| Cavdar2022 | * | * | * | * | - | * | * | * | * |
| He2022 | * | * | * | * | - | - | * | * | * |
| Li2022 | * | * | * | * | * | - | * | * | * |
| Liang2022 | * | * | * | * | * | - | * | * | * |
| Qu2022 | * | * | * | * | - | - | * | * | * |
| Shi2022 | * | * | * | * | - | * | * | * | * |
| Wang2022 | * | * | * | * | - | * | * | * | * |
| Wei2022 | * | * | * | * | - | * | * | * | * |
| Xu2022 | * | * | * | * | * | - | * | * | * |
| YeKeduz2022 | * | * | * | * | - | - | * | * | * |
| Yuan2022 | * | * | * | * | * | - | - | * | * |
| Chen2023 | * | * | * | * | * | - | * | * | * |
| Demircan2023 | * | * | * | * | * | - | * | * | * |
| Hannarici2023 | * | * | * | * | - | * | * | * | * |
| He2023 | * | * | * | * | - | - | * | * | * |
| Liu2023a | * | * | * | * | - | - | * | * | * |
| Liu2023b | * | * | * | * | * | - | * | * | * |
| Luo2023 | * | * | * | * | - | * | - | * | * |
| Nechita2023 | * | * | * | * | - | - | * | * | * |
| Shen2023 | - | * | * | * | * | * | * | * | * |
| Ucar2023 | * | * | * | * | - | - | * | * | * |
| Uzunoglu2023 | * | * | * | * | - | - | * | * | * |
| Wang2023 | - | * | * | * | * | * | * | * | * |
| Yang2023 | * | * | * | * | - | - | * | * | * |
| Zheng2023 | * | * | * | * | * | * | - | * | * |
| Aoyama2024 | * | * | * | * | * | - | * | * | * |
| Ba2024 | * | * | * | * | - | * | * | * | * |
| Chen2024 | * | * | * | * | - | - | * | * | * |
| Dong2024 | * | * | * | * | - | - | * | * | * |
| He2024 | * | * | * | * | * | - | * | * | * |
| Jing2024 | * | * | * | * | - | * | * | * | * |
| Wang2024 | * | * | * | * | - | - | * | * | * |
| Zeng2024 | * | * | * | * | - | * | * | * | * |
| Zhang2024 | * | * | * | * | - | - | * | * | * |
| Zhu2024 | * | * | * | * | - | - | * | * | * |
| Akdogan2025 | * | * | * | * | - | - | * | * | * |
| Du2025 | * | * | * | * | - | - | * | * | * |
| Guliyev2025 | * | * | * | * | * | - | * | * | * |
| Liu2025 | * | * | * | * | - | * | - | * | * |
| Mildanoglu2025 | * | * | * | * | * | - | * | * | * |
| Peker2025 | * | * | * | * | - | * | * | * | * |
| Shen2025 | * | * | * | * | - | * | * | * | * |
| Sun2025 | * | * | * | * | * | - | * | * | * |
| Udagawa2025 | * | * | * | * | - | - | * | * | * |
| Xie2025 | * | * | * | * | - | - | * | * | * |
| *indicates criterion met; - indicates significant of criterion not met. | | | | | | | | | |
|  | | | | | | | | | |
