# Supplementary material for: Systemic immune-inflammation index predicts survival in gastric cancer: a systematic review and meta-analysis
Source: Front Oncol. 2026 Oct 30;16:1953559. doi: 10.3389/fonc.2026.1953559 (PMC13759391; doi:10.3389/fonc.2026.1953559)
Supplement: Supplementary file 2 [file Table1.doc]

****Pubmed 145****

****((systemic immune-inflammation index) OR (SII)) AND (((((((((((Stomach Neoplasm) OR (Stomach Neoplasm)) OR (Gastric Neoplasms)) OR (Gastric Neoplasm)) OR (Cancer of Stomach)) OR (Stomach Cancers)) OR (Cancer of the Stomach)) OR (Gastric Cancer)) OR (Gastric Cancers)) OR (Stomach Cancer)) OR ("Stomach Neoplasms"[Mesh]))****

****EMBASE 163****

**((systemic immune-inflammation index or SII) and (Stomach Neoplasm or Stomach Neoplasm or Gastric Neoplasms or Gastric Neoplasm or Cancer of Stomach or Stomach Cancers or Cancer of the Stomach or Gastric Cancer or Gastric Cancers or Stomach Cancer or Stomach Neoplasms)).af.**

****Cochrane 4****

**((systemic immune-inflammation index or SII) and (Stomach Neoplasm or Stomach Neoplasm or Gastric Neoplasms or Gastric Neoplasm or Cancer of Stomach or Stomach Cancers or Cancer of the Stomach or Gastric Cancer or Gastric Cancers or Stomach Cancer or Stomach Neoplasms)).af.**

**Web of since 143**

**((systemic immune-inflammation index) OR (SII)) AND (((((((((((Stomach Neoplasm) OR (Stomach Neoplasm)) OR (Gastric Neoplasms)) OR (Gastric Neoplasm)) OR (Cancer of Stomach)) OR (Stomach Cancers)) OR (Cancer of the Stomach)) OR (Gastric Cancer)) OR (Gastric Cancers)) OR (Stomach Cancer)) OR (Stomach Neoplasms)) (Topic)**
